# Association of B Cells with Idiopathic Recurrent Pregnancy Loss: A Systematic Review and Meta-Analysis

**DOI:** 10.3390/ijms232315200

**Published:** 2022-12-02

**Authors:** Miguel Ângelo-Dias, Catarina Martins, Sara Simões Dias, Luís Miguel Borrego, Jorge Lima

**Affiliations:** 1 CHRC, NOVA Medical School, Faculdade de Ciências Médicas, NMS, FCM, Universidade NOVA de Lisboa, 1169-056 Lisboa, Portugal; 2 Immunology Department, NOVA Medical School, Faculdade de Ciências Médicas, NMS, FCM, Universidade NOVA de Lisboa, 1169-056 Lisboa, Portugal; 3 ciTechCare—Center for Innovative Care and Health Technology, Polytechnic of Leiria, 2411-901 Leiria, Portugal; 4 Department of Imunoallergy, LUZ SAÚDE, Hospital da Luz Lisboa, 1500-650 Lisboa, Portugal; 5 Department of Obstetrics and Gynecology, LUZ SAÚDE, Hospital da Luz Lisboa, 1500-650 Lisboa, Portugal

**Keywords:** B cells, idiopathic recurrent pregnancy loss, reproductive immunology, meta-analysis, MeSH

## Abstract

Recurrent pregnancy loss (RPL) affects 1–2% of women and is defined as having experienced two or more failed pregnancies. In almost 50% of cases, the causes are idiopathic (IRPL), but increasing evidence has suggested an immunological cause. B cells are known to provide crucial support for a successful pregnancy outcome. However, their involvement in the mechanisms underlying IRPL is still unclear. This systematic review and meta-analysis aimed to comprehensively summarise the existing evidence regarding the levels and profiles of B cells in IRPL. An extensive computerized search in PubMed/Medline, Embase, Scopus, and Web of Science databases was performed with no imposed limits. Two reviewers independently screened all retrieved studies, extracted all the data, and assessed the methodological quality. Disagreements were resolved by a third reviewer. From a total of 1125 retrieved studies, 19 studies were included in the systematic review, and 8 studies were quantitatively analysed. We highlight a potential association between women with IRPL and increased levels of endometrial B cells. In addition, the flow cytometry technique seems to be preferred over immunohistochemistry for identifying those differences, while further studies are necessary to clarify the role of B cells as an immunological risk factor for RPL.

## 1. Introduction

Recurrent pregnancy loss (RPL) is defined as experiencing two or more failed pregnancies prior to the 24th week of gestation and affects 1–2% of women [1,2]. Several causes and risk factors have been considered, including advanced maternal age, parental chromosomal abnormalities, uterine anatomical disorders, antiphospholipid syndrome, inherited thrombophilia, thyroid disorders, and even environmental factors. However, in nearly 50% of cases, the specific aetiology cannot be determined, and such cases are commonly referred to as unexplained or idiopathic RPL (IRPL) [3,4,5], with a significant psychological impact for the couples involved [6]. Many IRPL cases are treated empirically using several therapeutic strategies, including acetylsalicylic acid, progesterone, corticosteroids, low-molecular-weight heparin, intravenous immunoglobulin, lipid emulsion, leukocyte immune therapy, pre-implantation genetic screening, and tender loving care, but there is a paucity of high-quality evidence for the medical treatment of women with IRPL, with the exception of the use of progesterone [3,7].

Increasing experimental and clinical evidence suggests that immunological causes, such as immunological rejection or the presence of an unbalanced intrauterine immune homeostasis that is adverse for the embryo and pregnancy, could be significantly implicated in IRPL [8]. In fact, the maternal immune system plays a fundamental and challenging role during pregnancy. It ensures a state of tolerance for genetically foreign content while maintaining important protections against pathogens for both the mother and the developing foetus [9,10]. Looking at the maternal–foetal interface, we observed a maternal immune system acquiring distinctive features and articulating new functions and particular cell phenotypes, which suggest its commitment to assure the necessary processes taking place at these sites [11]. In this way, endometrial immune cells may contribute to the proper mechanisms for embryo implantation, survival and development. Therefore, is it of utmost importance to clarify the molecular mechanisms and specific cell types and cellular pathways involved in mediating endometrial receptivity.

B cells are a major component of the immune system. Recent studies have proven that during pregnancy, these cells undergo important adaptations, with physiological circulating B cell lymphopenia observed from mid-gestation onwards and a decreased presence of the more differentiated B cell subsets in the peripheral blood [12,13]. Moreover, altered B cell proportions and changes in their activation states were reported in different obstetric complications, including RPL, preterm birth, and pre-eclampsia [14,15,16]. In fact, differences in the peripheral B cell compartment have been previously observed in women with recurrent pregnancy losses. In particular, Kwak et al. [17] reported increased percentages of CD19^+^ B cells in pregnant women with RPL compared to multiparous pregnant normal controls. Later, Jablonowska et al. [18] also obtained similar results, with increased percentages and levels of peripheral B cells in first-trimester RPL pregnant women. In contrast, Darmochwal et al. [19] reported decreased percentages in CD19^+^ B cells when considering non-pregnant women with RPL compared to normal non-pregnant controls., while Ghafourian et al. [20] observed similar proportions of CD20^+^ B cells in both non-pregnant RPL and normal control women. Regarding the specific subsets of B cell compartments, it has been demonstrated that IL-10-producing regulatory B cells were decreased in a murine model of pregnancy loss compared to the normal pregnancy model, which present elevated levels in the first pregnancy trimester. Furthermore, it was shown that IL-10 administration and the transfer of IL-10-producing regulatory B cells in aborting animals could prevent foetal rejection [21]. In humans, increased regulatory B cell counts observed in the first trimester of pregnancy may also indicate the higher necessity to suppress possible unwanted immune maternal responses, thus protecting against pregnancy loss [22].

Interestingly, given the different leucocyte compositions of tissue, such as the decidua and the endometrium, which are rich in T cells, uterine NK cells (uNK), macrophages, and dendritic cells in comparison to peripheral blood stress, characterising these tissue types as only addressing circulating cells can be a limitation and is sometimes a biased perspective [23,24,25]. Thus, despite being considered a rare population with undetermined functions at these locations, B cells are present in the endometrium and they should be further characterised, particularly in pathological reproductive processes [23,26,27]. The initial characterisation of endometrial immune populations by Lachapelle and colleagues reported the presence of 6% of lymphocytic cells expressing the CD20 B cell marker in the endometrium of normal non-pregnant women [28]. Recently, similar proportions of CD19^+^ B cells using menstrual blood and term decidua parietalis samples of healthy non-pregnant women were also reported [29], while other studies presented different results [30,31]. Importantly, differences in endometrial B cells have also been demonstrated, with some studies reporting increased proportions of B cells in the endometrium of women with recurrent pregnancy losses [30,32].

The increasing number of studies characterising uterine B cells confirms the growing interest in the role of B cells in reproductive immunology. However, information on their potential involvement in the mechanisms underlying IRPL is still scarce and unclear. Hence, studying the role of B cells during IRPL deserves proper consideration. To congregate information and discuss what is known so far, we conducted a systematic review of the literature by addressing the association between idiopathic recurrent pregnancy loss and B cells, whether local or systemic.

## 2. Materials and Methods

This systematic review and meta-analysis aimed to identify and analyse human studies that assessed the role of B cell levels and profiles in non-pregnant women with recurrent pregnancy loss of unknown aetiology compared to non-pregnant healthy women.

This work was conducted and reported in line with the criteria of Preferred Reporting Items for Systematic Reviews and Meta-analyses (PRISMA) guidelines (Table S1) [33,34]. The updated methodology used in this systematic review is in accordance with the Cochrane Handbook of Systematic Reviews of Interventions [35] and is registered in the PROSPERO database (ID: CRD42020181418).

### 2.1. Search Strategy

An extensive computerised literature search was performed to retrieve studies that were included in this systematic review. The searches were performed in PubMed/Medline, Embase, Scopus, and Web of Science databases using database-specific subject heading terms and all variants in free-text words according to the specificities of each database (Table S2). Additionally, a supplementary search of the grey literature and of the reference sections of the selected studies and reviews was performed to identify any additional relevant missing publications that were not retrieved in the electronic search. No date or language limits were imposed on the search.

### 2.2. Eligibility Criteria and Study Selection

Study selection was independently performed by two individuals (MAD and JL) who screened the title and abstracts of all yielded articles from the queries according to the eligibility criteria displayed in Table 1.

Idiopathic RPL is defined as experiencing more than 2 or 3 failed pregnancies before 24 weeks of gestation in the absence of risk factors or commonly known causes of RPL, namely, uterine anatomic disorders, thyroid dysfunctions, inherited and acquired thrombophilia, and/or parental chromosomal disorders. Studies clearly not matching the eligibility criteria were excluded. No restrictions on geographical location, language of publication, or year of publication were applied, and all the non-primary literature was excluded, such as literature reviews, dissertations, theses, editorials, protocol studies, clinical guidelines, and abstracts or reports from meetings.

The full text of the remaining studies was analysed, and studies were included or discarded according to the eligibility criteria. Any disagreements were referred to a third reviewer (CM) to reach consensus. All decisions, including reasons for exclusion and the number of selected articles in each step, are recorded and depicted in a flow chart following the PRISMA 2020 guidelines. None of the review authors was blinded to journal titles or the study’s authors or institutions.

EndNote version 20 (bibliographic software) was used to store, organise, and manage all references arising from the literature search, including the management and removal of duplicates and scanning the titles/abstracts of all records.

### 2.3. Data Extraction and Quality Assessment

All relevant data were extracted from each selected study by two independent reviewers following a standardised piloting form methodology to minimise the risk of bias and to ensure full data extraction. Only information relevant to this review from studies assessing multiple outcomes and variables was collected. If applicable, the original study’s authors were contacted to clarify missing or unreported data. Any disagreements were referred to a third reviewer (CM) to reach consensus. The following data were extracted:

Identification of the study: title, authors, year of publication, journal title, country of origin, study design, and number of participants;

Participant characteristics: sample size, age, race, IRPL definition, clinical data of IRPL, and control groups;

Methodological features: sample characteristics, phase of sample collection, methodology used for B cell characterisation, and B cell markers;

Outcomes: B cell levels, B cell profiles, and number of miscarriages.

Methodological quality of each individual study was assessed independently by two reviewers (MAD and JL), using the NHLBI quality assessment tool for case–control studies. This scale evaluates 12 components of a study to determine its methodological quality. Therefore, evaluation criterion was answered either by “Yes”, “No”, “CD” (cannot determine), “NA” (not applicable), or “NR” (not reported). Subsequently, studies were graded as “Poor”, “Fair”, or “Good”. The level of bias will influence the evidence and results obtained in the systematic review; thus, studies deemed to be of “poor quality” were not included in the meta-analysis. Any disagreement between the two reviewers was referred to a third reviewer to reach consensus.

### 2.4. Data Synthesis

Meta-analyses were conducted using standardised mean differences (SMDs) and 95% confidence intervals (CIs) to allow a comparison of data from different instruments [36]. A random-effect model was used in the meta-analysis as it combines sampling errors and between-study variances to estimate the effect size [37]. To interpret the effect sizes, the following thresholds were used: <0.2 = trivial effect; 0.2–0.5 = small effect; 0.5–0.8 = moderate effect; >0.8 denoting large effects [38]. The statistical heterogeneity among studies was assessed using the I-squared (I^2^) value, which represents the percentage of variation across studies that is attributable to heterogeneities rather than chance [39]. We adopted the following thresholds: I^2^ = 25%: low heterogeneity; I^2^ = 50%: moderate heterogeneity; I^2^ = 75%: high heterogeneity [39]. Evident heterogeneities were investigated via subgroup analyses. Studies that did not report data as means ± SD were not suitable for inclusion in the meta-analysis.

All statistical analyses were conducted using statistical software R and using the package “meta” to perform the meta-analysis [40,41]. Statistical significance was defined as a *p*-value < 0.05.

## 3. Results

### 3.1. Study Selection

**Figure 1:**
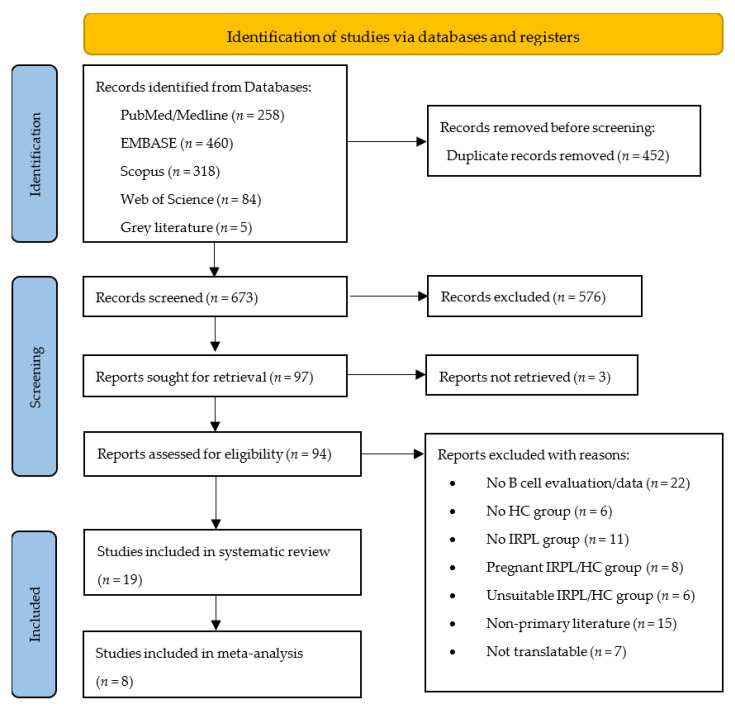
PRISMA 2020 flow diagram. PRISMA, Preferred Reporting Items for Systematic Reviews and Meta-analyses; HC, healthy control; IRPL, idiopathic recurrent pregnancy loss.

Using the aforementioned methodology, the database search yielded a total of 1125 studies, of which 452 were removed due to duplication. The abstract and title screening of 673 records revealed 576 studies that clearly did not meet the eligibility criteria, resulting in their exclusion. From the 97 records sought for retrieval, 94 full-text articles were obtained and critically analysed, leading to the exclusion of 75 studies due to ineligibility-related reasons. Finally, a total of 19 studies [17,19,20,30,31,32,42,43,44,45,46,47,48,49,50,51,52,53,54] were included in the qualitative analysis and 8 [20,32,42,43,46,48,50,54] were included in the quantitative analysis. Eleven studies were excluded from the meta-analysis because one had a high risk of bias [47] and ten [17,19,30,31,44,45,49,51,52,53] did not have the data required for performing the analysis. Figure 1 presents an outline of the study’s selection process.

### 3.2. Characteristics of Included Studies

All relevant data collected from each selected study are summarised in Table 2.

All studies were case–control observational studies conducted in 12 countries: China (*n* = 4) [45,46,53,54], Ireland (*n* = 2) [30,49], Spain (*n* = 2) [43,44], the US (*n* = 3) [17,47,48], and one study each from Egypt [42], Germany [31], Greece [50], Iran [20], Poland [19], the UK [51], Brazil [52], and Canada [32]. The RPL definition criteria used were not consistent among the studies, with 9 studies defining RPL as two or more pregnancy losses [30,31,43,46,47,49,50,52,54] and 10 studies using three or more pregnancy losses as the criterion for RPL [17,19,20,32,42,44,45,48,51,53]. Six studies did not report information regarding the number of miscarriages in the IRPL group [19,20,44,48,50,52].

As part of the inclusion criteria, all studies included and compared an IRPL group with a healthy control group. In six studies, the control group was composed of women with at least one live birth and no history of miscarriages [17,20,43,44,52,53], three studies included women with at least one live birth but sporadic cases of miscarriages in some [32,51,54], four studies included women with at least one successful pregnancy with no information regarding the previous history of miscarriages [19,42,45,46], two studies included a control group with no previous history of miscarriage but with no information regarding parity history [31,48], two studies included a control group with some miscarriages and some live births [30,49], and two studies did not report clinical information for the control group [47,50].

A total of 1386 IRPL women and 581 control women were included, with a mean sample size amongst studies of 73 (SD = 101; 9–411 range) in the IRPL group and 31 women (SD = 38; 8–179 range) in the control group. The studies used two different types of samples to characterise the immune compartment: peripheral blood (*n* = 12) [17,19,20,43,44,45,46,47,48,50,52,54] and endometrial biopsies (*n* = 7) [30,31,32,42,49,51,53]. Nine studies performed sample collection during the luteal phase of the menstrual cycle [30,31,32,42,46,49,51,52,53], while only one study performed sample collection during the follicular phase [44]. The remaining studies did not specify the phase of the menstrual cycle in which the samples were collected [17,19,20,43,45,47,48,50,54]. Flow cytometry was the preferred methodology used to assess the B cell population in studies (*n* = 16) [17,19,20,30,32,42,43,44,45,46,47,48,49,50,52,54], while immunohistochemistry was used in three studies [31,51,53]. B cells were identified mostly through CD19 lineage markers (*n* = 14) [17,19,30,31,43,44,45,46,47,48,49,50,52,54], followed by CD20 (*n* = 4) [20,32,42,53] and CD22 (*n* = 1) [51]. Additional markers were used in seven studies to identify specific B cell subsets, namely, CD5, IgD, CD27, and CD40 [17,19,43,44,47,48,50].

**Table 2:** Characteristics of included studies.

Reference	Country	*n* (Mean ± SD Age in Years)	iRPL Criteria	N of Miscarriages *	*n* (Mean ± SD Age in Years)	Obstetric History *	*n* of Studied Groups	Sample & Method	Sample Collection Phase	Markers	Results *
Alosh et al., 1998 [42]	Egypt	*n* = 20 (31.6 ± 4.2 yrs; 24–36 range)	3+	4.3 miscarriages (3–6 range)	*n* = 12 (33.2 ± 5.5; 28–38 range)	≥2 successful pregnancies.	2	EB|FC	Lutheal	CD20	↑ proportion of endometrial B cells in IRPL (17 ± 7%) compared to HC (5.5 ± 6%)—*p* < 0.05
Bohlmann et al., 2010 [31]	Germany	*n* = 25 (32.8 ± 5.6 yrs; 21–41 range)	2+	3.3 ± 1.17 miscarriages (2–6 range)	*n* = 10 (33.5 ± 4.3; 23–37 range)	No history of miscarriage.	2	EB|IHC	Lutheal	CD19	Similar CD19 staining score in IRPL (0.66 ± 0.64) and HC (0.38 ± 0.72).
Carbone et al., 2009 [43]	Spain	*n* = 36 (37 yrs, 30–43 range)	2+	2.89 miscarriages (2–7 range) 0.44 live-born babies (0–3 range)	*n* = 37 (37.0; 22–48 range)	≥1 live-born babies (1–3 range). No history of miscarriage.	5	PB|FC	Not specified	CD19, IgD, CD27 CD40, CD5	Similar % of B cells in IRPL (8 ± 3%, 7–8 95% CI) and HC (8 ± 4%, 7–10 95% CI). Similar B cell counts in IRPL (155 ± 76 cells/µL) and HC (159 ± 95 cells/µL). No significant differences regarding other B cell subsets.
Carbone et al., 2016 [44]	Spain	*n* = 24 (37 yrs; 32–43 range)	3+	NA	*n* = 37 (37,0)	History of a live child. No history of miscarriage.	3	PB|FC	Follicular	CD19, CD27, IgD	Similar total, naive (85 ± 52 vs. 99 ± 72 cells/µL) and class-switched memory B cell levels in IRPL and HC. ↑ levels of unswitched memory B cells in the IRPL group
Darmochwal-Kolarz et al., 2002 [19]	Poland	*n* = 14 (28.92 yrs; 25–34 range)	3+	NA	*n* = 18 (27.42; 26–35 range)	History of successful pregnancies.	2	PB|FC	Not specified	CD19^+^ CD19^+^ CD5^+^	↓ total B cell% in IRPL [8.5 (3.2–15.9)] ^#^ compared to HC [14.45 (10.9–20.7)] ^#^—*p* < 0.005. ↑ % of CD5^+^ B cell in IRPL [2.0 (0.7–5.9)] ^#^ compared to HC [0.9 (0.5–2.5)] ^#^—*p* < 0.05
Gao et al., 2014 [46]	China	*n* = 67 (30.28 ± 4.12 yrs)	2+	Total of 182 miscarriages	*n* = 22 (29.67 ± 3.29)	≥1 live birth.	3	PB|FC	Lutheal	CD19	Similar % of B cells in IRPL (13.19 ± 4.31) and HC (12.56 ± 3.36)—*p* = 0.232
Gao et al., 2021 [45]	China	*n* = 411 (30.22 ± 4.10 yrs)	3+	3.39 ± 0.66 miscarriages	*n* = 179 (30.82 ± 3.70)	≥1 live birth.	3	PB|FC	Not specified	CD19	Similar % of B cells in IRPL [11.0 (8.8–13.9)] ^$^ and HC [11.8 (10.4–13.0)] ^$^.
Ghafourian et al., 2014 [20]	Iran	*n* = 25 (20–35 yrs)	3+	NA	*n* = 25	History of a live child. No history of miscarriage.	2	PB|FC	Not specified	CD20	Similar % of B cells in IRPL (9.45 ± 0.71) and HC (11.34 ± 0.76).
Kwak et al., 1995 [17]	USA	*n* = 81 (33.6 ± 4.8 yrs)	3+	4.1 ± 1.4 miscarriages	*n* = 17 (36.5 ±7.0)	≥2 successful pregnancies. No history of miscarriage.	4	PB|FC	Not specified	CD19^+^ CD19^+^ CD5^+^	Similar % of B cells and CD5^+^ B cells in IRPL and HC.
Kwak et al., 1998 [47]	USA	*n* = 33 (34.0 yrs; 25–43 range)	2+	3.0 miscarriages (2–8 range)	*n* = 8	NA	2	PB|FC	Not specified	CD19^+^ CD19^+^ CD5^+^	The % of B cells was 12.8 ± 0.8 in the IRPL and 10.4 ± 1.4 in HC. The % of CD5^+^ B cells within total B cells was 43.7 ± 4.4 in the IRPL and 55.2 ± 13.0 in HC.
Lachapelle et al., 1996 [32]	Canada	1 ^ary^ IRPL: *n* = 11 (30 ± 4 yrs; 22–37 range) 2 ^ary^ IRPL: *n* = 9 (33 ± 2 yrs; 26–39 range)	3+	1 ^ary^ IRPL: 4 ± 1 miscarriages (3–6 range) 2 ^ary^ IRPL: 4 ± 1 miscarriages (3–5 range)	*n* = 15 (35.0 ± 4.0; 27–40 range)	≥1 live birth. 0.3 ± 0.5 miscarriages (0–1 range).	3	EB|FC	Lutheal	CD20	↑ % of endometrial B cells in IRPL (16.0 ± 8.0%) compared to HC (5.0 ± 6.0%)—*p* < 0.05. ↓ % of endometrial B cells in the IRPL group who had maintained an intact conceptus for extended periods compared to patients with continued miscarriages.
Mahmoud et al., 2001 [48]	USA	*n* = 10 (31.4 ± 2.2 yrs; 22–42 range)	3+	NA	*n* = 20 (29.5 ± 1.8, 20–43 range)	No history of RPL.	3	PB|FC	Not specified	CD19^+^ CD19^+^ CD5^+^	↓ % of B cells in RPL subjects (9.9 ± 1.1) compared to HC (13.9 ± 1.0)—*p* < 0.05.
Marron et al., 2019 [49]	Ireland	*n* = 121 (37.9 ± 4.0 yrs)	2+	Total of 320 miscarriages. Total of 47 live births.	*n* = 29 (35.2 ± 3.1)	Total of 4 miscarriages. Total of 10 live births.	5	EB|FC	Lutheal	CD19	↑ concentration endometrial of B cells in IRPL (79.6 cells/mg) compared to HC (48.8 cells/mg)—*p* = 0.002;
Marron et al., 2019 [30]	Ireland	*n* = 155 (38.0 ± 4.0 yrs)	2+	Total of 442 miscarriages. Total of 61 live births.	*n* = 35 (35.1 ± 2.9)	Total of 6 miscarriages. Total of 10 live births.	5	EB|FC	Lutheal	CD19	↑ % of endometrial B cells (within total CD45+ endometrial lymphocytes) in IRPL (0.77%) compared to HC (0.43%)—*p* < 0.001.
Psarra et al., 2001 [50]	Greece	*n* = 244 (26–39 yrs)	2+	NA	*n* = 44 (23–42 range)	NA	2	PB|FC	Not specified	CD19^+^ CD19^+^ CD5^+^	Similar % of B cells in IRPL (10.6 ± 3.8) and HC (11.4 ± 6.0). ↓ % of CD19+CD5+B cells within total lymphocytes in IRPL (0.4 ± 0.6) compared to HC (1.4 ± 0.8).
Quenby et al., 1999 [51]	UK	*n* = 22 (33.9 yrs; 20–41 range)	3+	4.4 miscarriages (3–17 range) 0.3 live births (0–2 range)	*n* = 9 (33.1; 24–41 range)	≥2 live births (2–4 range). 0.4 miscarriages (0–1 range).	2	EB|IHC	Lutheal	CD22	Similar median % of endometrial B cells within total cells in IRPL (0.18, 0–4 range) and HC (0, 0–0.8 range).
Souza et al., 2002 [52]	Brazil	*n* = 9	2+	NA	*n* = 9 (<40)	≥2 term pregnancies. No history of miscarriage.	2	PB|FC	Lutheal	CD19	↑ B cell counts in IRPL [215 (188–236) cells/mm^3^] ^$^ than in HC [182(151–185) cells/mm^3^] ^$^—*p* = 0.05
Zhao et al., 2020 [53]	China	*n* = 30 (35.40 ± 0.62 yrs)	3+	3.0 miscarriages (3–5 range)	*n* = 30 (29.47 ± 0.66)	≥1 live births. No history of spontaneous miscarriages.	2	EB|IHC	Lutheal	CD20	Similar B cell density in IRPL (0.5%, 0.2–2.5% range) and HC (0.4%, 0.1–2.2% range)—*p* = 0.0693
Zhu et al., 2015 [54]	China	*n* = 39 (28.3 ± 3.22 yrs)	2+	2.8 ± 0.6 miscarriages	*n* = 25 (26.8 ± 2.34)	Normal pregnancy history. 0.7 ± 0.34 miscarriages.	4	PB|FC	Not specified	CD19	Similar % of CD19+ B cells in IRPL (11.7 ± 3.31) and HC (11.7 ± 2.45)

IRPL, idiopathic recurrent pregnancy loss; PB, peripheral blood; FC, flow cytometry; EB, endometrial biopsy; IHC, immunohistochemistry; NA, not available; ↑ (increased); ↓ (decreased). * Mean ± SD, unless otherwise indicated; ^#^ median (min–max); ^$^ median (25th–75th percentile).

### 3.3. Methodological Quality

The methodological quality (risk of bias) of the included studies was assessed independently by MAD and JL, while any disagreement was discussed with a third author (CM) to reach consensus. The NHLBI Assessment Tool for case–control studies was used, which assesses the quality of a study through 12 questions that can be answered as yes, no, not applicable, not reported, or cannot determine, as shown in Table S3.

All studies explicitly defined the research question. With the exception of two studies [47,48], the study’s population was clearly specified and well defined, whereas only one study included a sample size justification [44]. Most of the studies had a low risk of bias when considering the group’s population recruitment, the specification and application of the inclusion and exclusion criteria, and the definition of cases and their differentiation from the controls. None of the studies performed a random selection of study participants, although this was not considered to be a fatal flaw, while most of the studies did not use concurrent controls. In none of the studies did the exposure (B cell evaluation) precede the outcome (miscarriages). Nevertheless, we consider that this question is not applicable in this context, since immune profile evaluation is usually recommended when women have already had two or more miscarriages with an unknown aetiology where immune dysregulation might be occurring. Finally, all studies measured the exposure in a consistent and valid manner, and most applied an adjustment for potential confounding variables in the statistical analysis. It was not possible to determine whether the exposure assessors were blinded to the case or control status of the participants.

Overall, 10 studies were considered to be at a low risk of bias and had good methodological quality [19,20,31,32,43,44,45,46,52,54]. Eight were considered to have fair methodological quality [17,30,42,48,49,50,51,53]. One study was deemed to be of poor quality [47]; thus, it was not included in the meta-analysis due to its potential high risk of bias.

### 3.4. Results of Individual Studies and Meta-Analyses

Considering all included studies, a majority (*n* = 11) reported no significant differences between the proportion or concentration of total B cells in women with IRPL compared to HC women [17,20,31,43,44,45,46,50,51,53,54]. Seven studies reported statistically significant differences between groups [19,28,30,42,48,49,52], and one study did not perform statistical analyses on B cell data [47].

**Figure 2:**
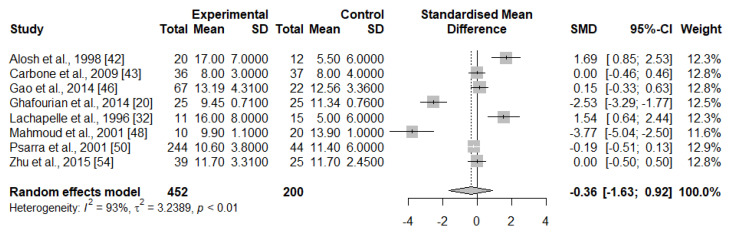
Random-effect meta-analysis for the levels of B cells in IRPL and HC women.

We pooled data from eight studies (*n* = 652 women) and observed a non-significant tendency towards lower proportions of total B cells in the IRPL group (SMD = −0.36 [95% CI, −1.63–0.92]), with a high heterogeneity among the included studies (I^2^ = 93%) (Figure 2).

Subgroup analyses were carried out to explore possible causes of heterogeneity according to the type of sample used in the studies: peripheral blood or endometrial biopsies.

**Figure 3:**
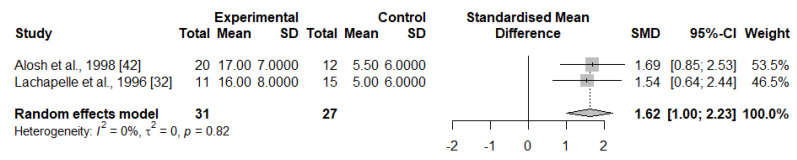
Random-effect meta-analysis for the levels of endometrial B cells in IRPL and HC women.

From the seven studies using endometrial biopsies, all those (*n* = 4) characterising the B cell compartment with flow cytometry reported significant differences towards increased percentages or concentrations of total B cells in IRPL compared to the HC group [30,32,42,49]. The remaining three studies used immunohistochemistry to evaluate B cell compartment and did not find significant differences between groups [31,51,53]. We pooled data from two studies (*n* = 58 women) that reported B cell data in endometrial biopsies. Overall, there were higher proportions of total B cells in the IRPL group (SMD = 1.62 [95% CI, 1.00–2.23]; *p* < 0.001, I^2^ = 0%) (Figure 3).

**Figure 4:**
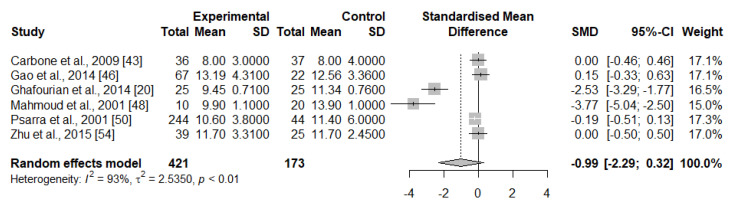
Random-effect meta-analysis for the levels of circulating B cells in IRPL and HC women.

From the 12 studies using peripheral blood samples, a majority (*n* = 8) reported similar percentages of B cells between groups [17,20,43,44,45,46,50,54], while two reported significantly lower percentages of total B cells in IRPL women compared to HC [19,48]. These observations were not shared by Souza et al., 2002 [52], who reported increased B cell counts in IRPL compared to HC women. We pooled data from six studies (*n* = 594 women) that evaluated B cells in peripheral blood. Overall, the analyses revealed no statistically significant associations between peripheral B cell levels and IRPL (SMD = −0.99 [95% CI, −2.29–0.32]; *p* > 0.05, I^2^ = 93%) (Figure 4).

Regarding the evaluation of specific B cell subsets in the included studies, one study reported increased levels of circulating unswitched memory B cells in IRPL compared to the HC group [44], one study reported increased percentages of circulating CD5^+^ B cells within total lymphocytes in IRPL compared to the HC group [19], and one study reported decreased percentages of circulating CD5^+^ B cells within total lymphocytes in IRPL compared to the HC group [50]. Due to the small number of studies addressing specific B cell subsets, a meta-analysis was not feasible for this subgroup.

## 4. Discussion

Reproductive failure is a pregnancy-related complication that represents a significant concern for human reproduction. In this context, immunological abnormalities have been implicated in many female reproductive pathologies, including recurrent pregnancy loss [8,55]. Unlike other immune compartments [56,57,58], the current scarce, sparse, and methodologically unstandardised available data on the involvement of B cells (their levels and profiles) as a risk factor in the aetiology of recurrent pregnancy loss represent and point to the challenge in obtaining consistent conclusions on this matter. Therefore, it is of utmost interest to summarise, in a transparent, structured, and organised manner, the existing literature evaluating B cell compartments in women with IRPL. To the best of our knowledge, this is the first systematic review with a meta-analysis that addresses studies evaluating the involvement of B cells in non-pregnant women with IRPL compared to non-pregnant healthy controls. In this review, a total of 19 studies were selected in the qualitative analysis, and from these, 8 were included in the meta-analysis.

Here, we highlight the potential association between women with IRPL and increased levels of endometrial B cells (compared to HC women). In contrast, no such associations were found when considering peripheral B cells, which is not surprising, since distributions and functions of immune populations, including B cells, are not equal when considering different tissue types [59,60]. Unfortunately, insufficient studies evaluating specific B cell subsets were retrieved. Interestingly, although only two studies characterising endometrial B cells were quantitatively analysed, all retrieved studies evaluating endometrial B cells with flow cytometry (*n* = 4) reported significantly increased levels of endometrial B cells in IRPL compared to the HC group, whereas studies using immunohistochemistry did not report differences between groups. This might indicate that the endometrial characterisation of B cells is preferred over peripheral characterisation to identify differences between these subsets in IRPL women. In addition, the use of flow cytometry as the methodological tool to characterise those differences seems to be advantageous compared to immunohistochemistry.

Our systematic review has several strengths. The predefined methodology used in this review was based on the Cochrane Handbook for Systematic Reviews of Interventions [35], and we conducted our review using a prospectively registered protocol. In addition, we employed a comprehensive and extensive search strategy using the most representative electronic bibliographic databases for biomedical research. No restrictions were applied to the search so that all possibly relevant studies were retrieved, and the risk of bias was evaluated independently by two individuals. Finally, a meta-analysis was performed using the random-effect model to pool the data as much as possible.

However, we also acknowledge some limitations of this review. First, although we were able to identify a large number of studies reporting the levels of B cells in IRPL women, there were significant heterogeneities among them, namely, in the criteria for the definition of RPL, the lack of or limited clinical reported information from the included groups, particularly for the control group, the large number of countries in which the selected studies were conducted, and the distinct sample types and methodologies used to characterise B cell data. Importantly, our significant findings should be interpreted with caution, as some of our meta-analysis included data from a reduced number of studies, which—although valid—might limit the value of pooling data. In fact, although it is a limitation of the included studies, only a small portion of the selected studies (*n* = 8) reported B cell data suitable for inclusion in the meta-analysis. Other conclusions or more robust findings might have been identified if all studies were included in the meta-analysis. Due to the small number of studies, we were unable to adjust for some important confounding variables, such as age, ethnicity, country of origin and number of previous miscarriages, which could affect our findings. In addition, we did not consider formal tests for publication bias, since fewer than 10 studies were included in the meta-analysis [35].

Finally, although this review emphasises that increased levels of endometrial B cells might play a role during the processes of miscarriage, it is still unclear whether these higher levels of B cells are causes or consequences of the RPL. To further explore this causality, it would be more accurate to compare women with a known cause for the RPL as a control group with women with IRPL. Thus, if B cells are equally increased in this control group, then B cells may likely represent a consequence of miscarriages rather than the cause.

We have identified a systematic review published in HROpen journal that is somewhat similar to our present work [61]. However, in this review, the authors did not specifically analyse the RPL of unknown origins or analyse both circulating and local B cell compartments in the IRPL. In addition, this review includes data from both pregnant and non-pregnant RPL women; thus, the results obtained in those studies might reflect pregnancy-induced changes in B cell populations, rather than changes associated with the pathology of interest. Importantly, in this systematic review, the authors were unable to perform a quantitative synthesis, so the conclusiveness of the presented evidence in that review is unclear. Nevertheless, we recognise that the existing heterogeneity among the available studies (either due to differences in the methodology, study design, or selection criteria) on this matter represents a problem in the attempt to provide solid conclusions regarding the role of B cells in IRPL.

Overall, although there is an apparent association between increased endometrial B cells and IRPL, their role and levels in the development of this condition are not well understood. The use of flow cytometry could be a valuable tool to evaluate different endometrial B cell phenotypes in IRPL and to further explore this association. Nevertheless, further studies are necessary to clarify the role of B cells as an immunological risk factor for RPL, and we expect that this review will provide clues and important data to stimulate further research on this matter.

## Figures and Tables

**Table 1:** Eligibility criteria for study selection.

Inclusion Criteria	Exclusion Criteria
Studies on living humans	Animal studies
Women of reproductive age (18–45 years)	Women with current pregnancy
Recurrent pregnancy loss of unknown aetiology	Studies not reporting B cell levels
IRPL group with at least two consecutives miscarriages	Genetic studies
B cell compartment evaluation	No primary research
	Case-report studies

IRPL, idiopathic recurrent pregnancy loss.

## Data Availability

Not applicable.

## Supplementary Materials

The following supporting information can be downloaded at https://www.mdpi.com/article/10.3390/ijms232315200/s1. Table S1: Preferred Reporting Items for Systematic Reviews and Meta-analyses checklist; Table S2: Retrieval search strategy; Table S3: Methodological quality assessment of selected studies.
