# Supplementary material for: Association of B Cells with Idiopathic Recurrent Pregnancy Loss: A Systematic Review and Meta-Analysis
Source: Int J Mol Sci. 2022 Dec 2;23(23):15200. doi: 10.3390/ijms232315200 (PMC9736675; doi:10.3390/ijms232315200)
Supplement: Supplementary file 1 [file ijms-23-15200-s001.zip › Table S2 - Retrieval Search Strategy.pdf]

Table S2. Retrieval search strategy

QUERY	SEARCH
**PubMed/Medline**	
#1	(abortion, habitual[MeSH Terms]) OR (habitual abortion) OR (recurrent miscarriage) OR (recurrent abortion) OR (recurrent pregnancy loss) OR (spontaneous abortion) OR (repeated abortion) OR (repeated miscarriage) OR (repetitive miscarriage) OR (repetitive abortion) OR (recurrent reproductive failure) OR (pregnancy loss)
#2	(B lymphocyte[MeSH Terms]) OR (B-cell) OR (B-lymphocyte) OR (B cell) OR (B lymphocyte) OR (CD19)
#3	#1 AND #2
**EMBASE**	
#1	'recurrent abortion'/exp OR 'recurrent pregnancy loss'/exp OR 'reproductive failure'/exp OR 'spontaneous abortion'/exp OR 'pregnancy loss'/exp OR 'recurrent miscarriage'/exp OR 'reproductive failure' 'recurrent abortion' OR 'habitual abortion' OR 'repeated abortion' OR 'successive abortion' OR 'spontaneous abortion' OR 'repeated miscarriage' OR 'repetitive miscarriage' OR 'recurrent pregnancy loss' OR 'pregnancy loss' OR 'recurrent miscarriage' OR 'repetitive abortion'
#2	'B lymphocyte'/exp OR 'B cell' OR 'B lymphocyte' OR 'B-cell' OR 'B-lymphocyte' OR 'CD19'
#3	#1 AND #2
**Scopus**	
#1	TITLE-ABS-KEY("habitual abortion") OR TITLE-ABS-KEY("recurrent miscarriage") OR TITLE-ABS-KEY("recurrent abortion") OR TITLE-ABS-KEY("recurrent pregnancy loss") OR TITLE-ABS-KEY("spontaneous abortion") OR TITLE-ABS-KEY("repeated abortion") OR TITLE-ABS-KEY("repeated miscarriage") OR TITLE-ABS-KEY("repetitive miscarriage") OR TITLE-ABS-KEY("repetitive abortion") OR TITLE-ABS-KEY("reproductive failure") OR TITLE-ABS-KEY("pregnancy loss") OR TITLE-ABS-KEY("successive abortion")
#2	TITLE-ABS-KEY("B lymphocyte") OR TITLE-ABS-KEY("B-cell") OR TITLE-ABS-KEY("B-lymphocyte") OR TITLE-ABS-KEY("B cell") OR TITLE-ABS-KEY("CD19")
#3	#1 AND #2
**Web of Science**	
#1	TS=("habitual abortion" OR "recurrent miscarriage" OR "recurrent abortion" OR "recurrent pregnancy loss" OR "spontaneous abortion" OR "repeated abortion" OR "repeated miscarriage" OR "repetitive miscarriage" OR "repetitive abortion" OR "reproductive failure" OR "pregnancy loss" OR "successive abortion") AND TS=("B lymphocyte" OR "B-cell" OR "B-lymphocyte" OR "B cell" OR "CD19")

MeSH, Medical Subject Headings.
